# Oxidative Stress-Responsive Apoptosis Inducing Protein (ORAIP) Plays a Critical Role in High Glucose-Induced Apoptosis in Rat Cardiac Myocytes and Murine Pancreatic β-Cells

**DOI:** 10.3390/cells6040035

**Published:** 2017-10-18

**Authors:** Takako Yao, Tsutomu Fujimura, Kimie Murayama, Ko Okumura, Yoshinori Seko

**Affiliations:** 1 Division of Cardiovascular Medicine, Institute for Adult Diseases, Asahi Life Foundation, Tokyo 103-0002, Japan; apr16_taco@yahoo.co.jp; 2 Laboratory of Bioanalytical Chemistry, Tohoku Medical and Pharmaceutical University, Sendai 981-0905, Japan; fujitsu@juntendo.ac.jp; 3 Division of Proteomics and Biomolecular Science, BioMedical Research Center, Graduate School of Medicine, Juntendo University, Tokyo 113-8421, Japan; murayama@juntendo.ac.jp; 4 Department of Biofunctional Microbiota, Graduate School of Medicine, Juntendo University, Tokyo 113-8421, Japan; kokumura@juntendo.ac.jp

**Keywords:** apoptosis, cardiac myocytes, diabetes mellitus (DM), high glucose, oxidative stress, oxidative stress-responsive apoptosis inducing protein (ORAIP), pancreatic β-cells

## Abstract

We previously identified a novel apoptosis-inducing humoral factor in the conditioned medium of hypoxic/reoxygenated-cardiac myocytes. We named this novel post-translationally-modified secreted-form of eukaryotic translation initiation factor 5A Oxidative stress-Responsive Apoptosis-Inducing Protein (ORAIP). We confirmed that myocardial ischemia/reperfusion markedly increased plasma ORAIP levels and rat myocardial ischemia/reperfusion injury was clearly suppressed by neutralizing anti-ORAIP monoclonal antibodies (mAbs) in vivo. In this study, to investigate the mechanism of cell injury of cardiac myocytes and pancreatic β-cells involved in diabetes mellitus (DM), we analyzed plasma ORAIP levels in DM model rats and the role of ORAIP in high glucose-induced apoptosis of cardiac myocytes in vitro. We also examined whether recombinant-ORAIP induces apoptosis in pancreatic β-cells. Plasma ORAIP levels in DM rats during diabetic phase were about 18 times elevated as compared with non-diabetic phase. High glucose induced massive apoptosis in cardiac myocytes (66.2 ± 2.2%), which was 78% suppressed by neutralizing anti-ORAIP mAb in vitro. Furthermore, recombinant-ORAIP clearly induced apoptosis in pancreatic β-cells in vitro. These findings strongly suggested that ORAIP plays a pivotal role in hyperglycemia-induced myocardial injury and pancreatic β-cell injury in DM. ORAIP will be a biomarker and a critical therapeutic target for cardiac injury and progression of DM itself.

## 1. Introduction

Cardiovascular injury involved in DM is known to be mainly due to coronary artery disease with myocardial ischemia/reperfusion as well as direct metabolic cardiac myocyte injury by hyperglycemia. Although oxidative stress has been implicated in both mechanisms, the precise mechanism of the latter remains unclear. We previously identified a novel apoptosis-inducing humoral factor, in a conditioned medium from cardiac myocytes subjected to hypoxia/reoxygenation, to be tyrosine-sulfated and more hypusinated secreted form of eukaryotic translation initiation factor 5A (eIF5A) [1]. We found that eIF5A undergoes 69th tyrosine-sulfation in the *trans*-Golgi and is rapidly secreted from cardiac myocytes in response to hypoxia/reoxygenation, then, induces apoptosis by acting as a pro-apoptotic ligand. The apoptosis of cardiac myocytes induced by hypoxia/reoxygenation was suppressed by anti-eIF5A neutralizing mAbs in vitro. Myocardial ischemia/reperfusion (but not ischemia only) rapidly and markedly increased plasma levels of eIF5A, which returned to the control levels within 60 min. In vivo treatment with anti-eIF5A neutralizing mAbs significantly reduced myocardial ischemia/reperfusion injury. These results demonstrated that a novel post-translationally modified secreted form of eIF5A is a specific biomarker and a critical therapeutic target for oxidative stress-induced cell injury. We named this novel tyrosine-sulfated secreted form of eIF5A Oxidative stress-Responsive Apoptosis Inducing Protein (ORAIP) [1]. We confirmed that ORAIP is specifically secreted in response to the oxidative stresses including ischemia/reperfusion, hypoxia/reoxygenation, ultraviolet-irradiation, ionizing radiation, cold/warm-stress (heat shock), and blood acidification [1,2], then acts as a pro-apoptotic ligand to induce apoptosis in target cells such as cardiac myocytes. In addition to these acute oxidative stresses, we also found that the plasma levels of ORAIP were markedly elevated in patients with chronic diseases such as chronic kidney disease and atrial fibrillation in which oxidative stress plays a critical role in the pathogenesis involved [3,4]. Furthermore, evidence has accumulated that loss of pancreatic β-cells through hyperglycemia-induced apoptosis plays a critical role in the development and progression of type 2 DM [5,6]. The purpose of this study was to investigate the pathogenesis of hyperglycemia-induced direct cell injury of cardiac myocytes as well as pancreatic β-cells involved in DM. First, we analyzed plasma levels of ORAIP in DM model rats during diabetic phase and non-diabetic phase. Second, we investigated the role of ORAIP in high glucose-induced apoptosis in cardiac myocytes in vitro. We also analyzed whether ORAIP induces apoptosis in pancreatic β-cells in vitro.

## 2. Materials and Methods

**Animals and culture cells:** This study was carried out in accordance with the Guide of The Japanese Association of Laboratory Animal Facilities of National University Corporations and with approval of institutional animal care committee. We used Hos:ZFDM*-Lepr^fa^* (ZFDM) male rats as type 2 DM model rats [7]. They were fed with high fat diet (58Y1; PMI Nutrition International, USA). Primary cultures of ventricular cardiac myocytes were prepared from neonatal rats as described elsewhere [8]. Briefly, heart ventricles were aseptically removed from neonatal Wistar rats, minced in calcium-free phosphate buffered-saline (PBS), and digested with 0.125% trypsin-ethylenediaminetetraacetic acid (EDTA) in PBS. The isolated cardiac myocytes were washed in Dulbecco’s Modified Eagle Medium (DMEM) containing 10% fetal calf serum (FCS) and 100 mg/mL glucose, dispersed into plastic dishes for 1 h to separate the fibroblasts, and removed to new gelatin-coated culture dishes. They were cultured for 36 h until they were confluent, then subjected to high concentration (55.5 mM) of glucose with mouse IgG or anti-ORAIP mAb (0.05 g/L). Murine pancreatic β-cell line (MIN6) was established from insulinomas obtained by targeted expression of the simian virus 40 T antigen gene in transgenic mice. MIN6 cells produce insulin and T antigen and have morphological characteristics of pancreatic β-cells. MIN6 cells exhibit glucose-inducible insulin secretion comparable with cultured normal murine islet cells [9], and cultured in DMEM containing 15% FCS and (25.0 mM) glucose.

**Anti-eIF5A mAbs:** A mouse anti-eIF5A mAb (clone YSP5-45-36) was generated against human eIF5A peptides (amino acid residues 44 to 72, which includes the hypusination site and 69th tyrosine sulfation site, coupled to KLH). Another mouse anti-eIF5A mAb (clone YSPN2-74-18) was generated against human eIF5A peptides (amino acid residues 7 to 33, near N-terminal region, coupled to KLH) as described previously [1].

**Enzyme-linked immunosorbent assay (ELISA):** The sandwich ELISA was performed with YSPN2-74-18 as a capture antibody fixed on the wells of microtiter strips. Plasma samples were pipetted into the wells and incubated. After washing, horseradish peroxidase (HRP)-labeled YSP5-45-36 was added as a detection antibody and incubated. After washing, color development was carried out by addition of a substrate solution, as described previously [1].

**Immunofluorescence:** Immunofluorescent staining of ORAIP was performed using Tyramide Signal Amplification (TSA) technology for fluorescence (TSA^TM^ Biotin System, PerkinElmer, Waltham, MA, USA). Double-immunostaining for cardiac myosin was performed as described elsewhere [10]. The cells were incubated with an anti-cardiac myosin mAb (clone CMA19 [11]) followed by incubation with tetramethylrhodamine isothiocyanate (TRITC)-labeled anti-mouse IgG. For double-immunostaining of cultured MIN6 cells for ORAIP and insulin, the cells were fixed in acetone for 5 min, and were first incubated with mouse anti-insulin mAb (L6B10; Cell Signaling Technology, Danvers, MA, USA) followed by incubation with TRITC-labeled anti-mouse IgG. Second, the cells were incubated with HRP-labeled anti-ORAIP mAb (YSP5-45-36), followed by incubation with biotinylated-Tyramide, and then with fluorescein-avidin D.

**TUNEL staining and cardiac myosin immunostaining:** We used the In Situ Apoptosis Detection Kit (TAKARA BIO Inc., Kusatsu, Japan) followed by diaminobenzidine (DAB) reaction (brown color) for TUNEL staining. For cardiac myocytes, additionally, the cells were incubated with an anti-cardiac myosin mAb (CMA19) followed by alkaline phosphatase-labeled anti-mouse IgG (Santa Cruz Biotechnology, Dallas, TX, USA). The cells were then reacted with an alkaline phosphatase substrate (alkaline phosphatase substrate kit III, Vector Laboratories, Burlingame, CA, USA) to produce a blue reaction product.

## 3. Results

### 3.1. Hyperglycemia Markedly Increases Plasma ORAIP Levels

**Figure 1:**
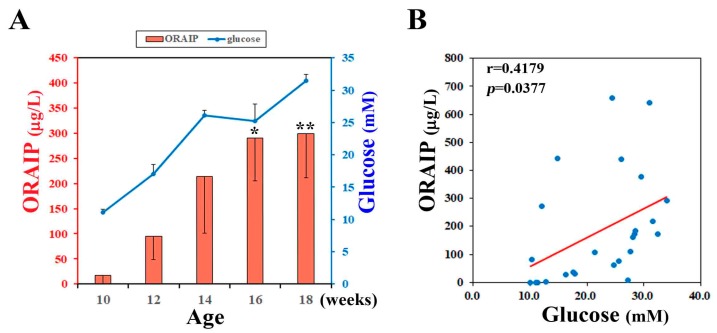
(**A**) Plasma levels of ORAIP (Oxidative stress-Responsive Apoptosis Inducing Protein) (red bar graph, left Y-axis) and those of glucose (blue line graph, right Y-axis) in ZFDM rats (mean ± SE, *n* = 5, * *p* = 0.0257 and ** *p* = 0.0177 vs. 10-week-old; paired *t*-test); (**B**) Correlation between plasma levels of ORAIP and glucose. There was a significant positive correlation (*r* = 0.418, *p* = 0.0377). A red line indicates the regression line.

To investigate the effect of hyperglycemia on plasma levels of ORAIP, we measured plasma levels of non-fasting glucose and ORAIP in DM model (ZFDM) rats during pre-diabetic to diabetic phase (at 10, 12, 14, 16, 18-week-old). The (mean ± SE, *n* = 5) plasma ORAIP levels (16.7 ± 16.3 μg/L) (Figure 1A; red bars) at pre-diabetic phase (10-week-old), when plasma glucose levels were (11.11 ± 0.48 mM), were slightly increased compared with normal control range (<10.0 μg/L) [1]. Then, plasma ORAIP levels began to increase as plasma glucose levels (Figure 1A; blue line) increased, and were significantly increased at 16–18 weeks-old (299.8 ± 88.4 μg/L) compared with 10-week-old (Figure 1A). Figure 1B shows the correlation between plasma levels of ORAIP and glucose in these 5 rats. There was a significant positive correlation (*r* = 0.418, *p* = 0.0377) between them, strongly suggesting that hyperglycemia plays a critical role in increasing plasma ORAIP levels.

### 3.2. High Glucose Induces Expression of ORAIP in Cultured Cardiac Myocytes

**Figure 2:**
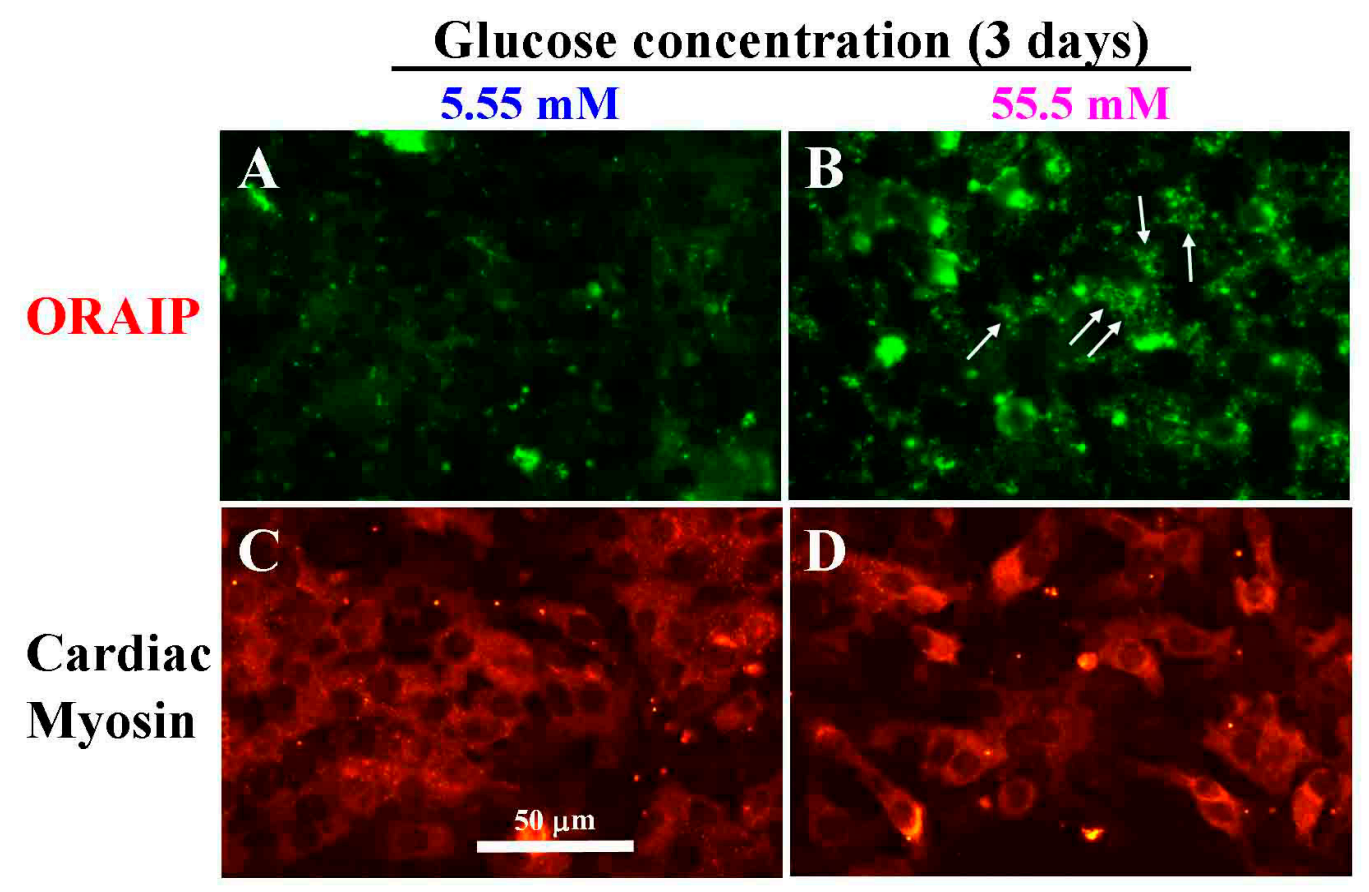
High glucose induces expression of ORAIP in cardiac myocytes in vitro. (**A**,**C**) Myocytes under normal concentration (5.55 mM) of glucose for three days; (**B**,**D**) Myocytes under high concentration (55.5 mM) of glucose for three days. (**A**,**B**) stained with anti-ORAIP mAb and labeled with fluorescein; (**C**,**D**) which correspond to A and B, respectively, show the staining pattern specific for cardiac myosin and labeled with TRITC. Bar, 50 μm.

Next, to investigate the direct effect of hyperglycemia on cardiac myocytes, especially the expression of ORAIP in cardiac myocytes, we examined whether high glucose induces the expression of ORAIP in cardiac myocytes in vitro. We cultured cardiac myocytes under high glucose concentration (55.5 mM) for 72 h and analyzed the expression of ORAIP in cardiac myocytes by double-immunostaining for ORAIP and cardiac myosin. As shown in Figure 2A,C, cardiac myocytes cultured under normal glucose concentration (5.55 mM) expressed only low or almost no expression of ORAIP. Whereas many cardiac myocytes cultured under high concentration (55.5 mM) of glucose clearly expressed ORAIP in granule-like morphology, suggesting that abundant ORAIP molecules were being secreted.

### 3.3. Anti-ORAIP mAb Mostly Suppressed High Glucose-Induced Apoptosis in Cultured Cardiac Myocytes

**Figure 3:**
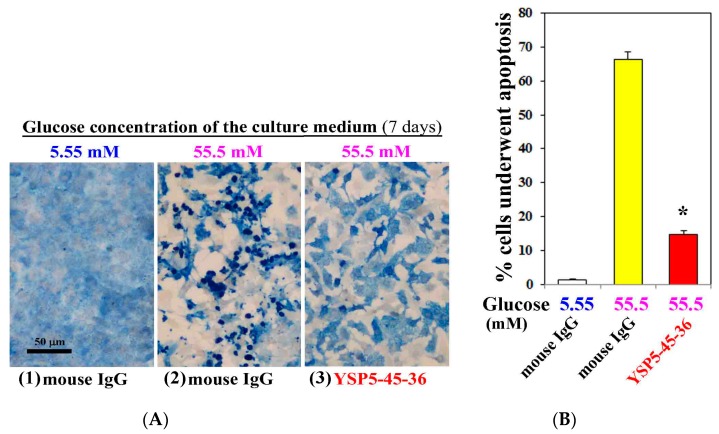
Anti-ORAIP mAb mostly suppressed high glucose-induced apoptosis in cultured cardiac myocytes in vitro. (**A**) Induction of apoptosis in cardiac myocytes as determined by TUNEL staining (brown) and cardiac myosin immunostaining (blue). Representative images at seven days by treatment with high glucose and mouse IgG or anti-ORAIP mAb (YSP5-45-36) (0.05 g/L). Bar, 50 μm; (**B**) The percentage of apoptotic cardiac myocytes, as determined by TUNEL staining, induced by treatment with high glucose and mouse IgG or anti-ORAIP mAb (YSP5-45-36). The data are expressed as the mean ± SE. (*n* = 4 for each). * *p* < 0.0001 vs. Glucose (55.5 mM) with mouse IgG (Dunnett’s multiple comparison test).

To investigate the role of hyperglycemia in the cardiac myocyte injury, we examined apoptosis induction of cardiac myocytes cultured under high concentration (55.5 mM) of glucose for 7 days by TUNEL staining. We also analyzed the effect of anti-ORAIP neutralizing mAb on the induction of apoptosis by high concentration (55.5 mM) of glucose. As shown in Figure 3, only minimal apoptosis was induced under normal concentration (5.55 mM) of glucose (Figure 3(A1),B). Whereas high concentration (55.5 mM) of glucose with control mouse IgG (0.05 g/L) extensively induced apoptosis in cardiac myocytes (66% of whole cells), which was 77% suppressed by treatment with an anti-ORAIP neutralizing mAb (YSP5-45-36, 0.05 g/L) (Figure 3(A2,3),B). This indicates that high glucose-induced apoptosis in cardiac myocytes was mostly mediated by ORAIP secreted by cardiac myocytes in an autocrine fashion. As shown in Figure 3(A2,3), high concentration (55.5 mM) of glucose induced morphological changes of cardiac myocytes, which was thought to be due to high osmotic pressure. However, treatment with an anti-ORAIP neutralizing mAb revealed that high glucose itself induced morphological changes but not apoptosis in cardiac myocytes.

### 3.4. MIN6 Cells Strongly Express ORAIP and Recombinant-ORAIP Induces Apoptosis in MIN6 Cells In Vitro

**Figure 4:**
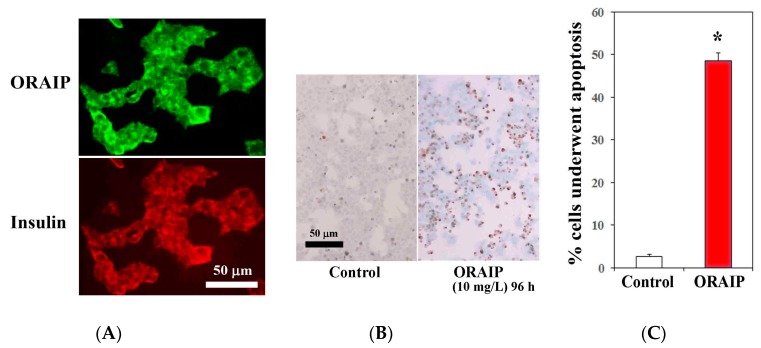
Expression of ORAIP in murine pancreatic β-cells (MIN6) and recombinant-ORAIP induces apoptosis in MIN6 cells in vitro. (**A**) Immunofluorescent localization of ORAIP proteins in cultured murine pancreatic β-cells (MIN6) under (25.0 mM) concentration of glucose. (**B**) Induction of apoptosis in MIN6 cells by treatment with recombinant-ORAIP (10 mg/L) for 96 h as determined by TUNEL staining. Bar, 50 μm. (**C**) The percentage of apoptotic MIN6 cells treated by recombinant-ORAIP. The data are expressed as the mean ± SE. (*n* = 4 for each). * *p* = 0.000059 vs. Control (Welch’s *t*-test).

Next, to investigate the role of ORAIP in hyperglycemia-induced pancreatic β-cell injury, first, we examined the expression of ORAIP by double immunostaining for ORAIP and insulin. As shown in Figure 4A, MIN6 cells strongly expressed both ORAIP and insulin in granule-like morphology under high concentration (25.0 mM) of glucose, strongly suggesting that the pancreatic β-cells secrete ORAIP as well as insulin in response to hyperglycemia. Findings of the present study strongly suggest that cells such as cardiac myocytes and pancreatic β-cells secrete ORAIP in response to hyperglycemia, which in turn increases plasma levels of ORAIP. Next, to investigate whether elevated plasma levels of ORAIP induces apoptosis of pancreatic β-cells, we analyzed apoptosis induction of MIN6 cells by recombinant-ORAIP in vitro. As shown in Figure 4B,C, only small percentage (less than 3%) of MIN6 cells underwent apoptosis under (25.0 mM) concentration of glucose. Treatment with recombinant-ORAIP (10 mg/L) markedly increased the percentage of apoptotic MIN6 cells up to 49% in 96 h. Taken together, it is suggested that hyperglycemia-induced secretion of ORAIP from pancreatic β-cells facilitates the apoptosis in β-cells in an autocrine fashion as in cardiac myocytes. Thus, ORAIP may play a pivotal role in the pathogenesis of type 2 DM progression.

## 4. Discussion

Oxidative stress has been implicated in the pathogenesis of cardiovascular, neuronal, and ophthalmic complications as well as insulin resistance in DM. However, the precise mechanism involved is still elusive and remains to be elucidated. Although hyperglycemia-induced production of reactive oxygen species (ROS) in various cells including cardiac myocyes and pancreatic β-cells was reported to be the mechanism involved [12,13], large scale antioxidants (such as vitamin E and C) clinical trials have proved to be disappointing to reduce cardiovascular diseases in humans [14,15,16,17]. This raises a possibility that there is some unknown mechanism other than ROS that mediates oxidative stress-induced cell injury.

In this study, we clearly showed that plasma ORAIP levels were markedly elevated during the diabetic phase in DM model rats and there was a significant positive correlation between plasma levels of glucose and ORAIP, strongly suggesting that hyperglycemia plays a critical role in increasing plasma ORAIP levels, which in turn causes cardiovascular injury as well as islet cell injury. High glucose significantly induced the expression of ORAIP in cardiac myocytes, resulting in massive apoptosis in cardiac myocytes in an autocrine fashion in vitro. This was mostly suppressed by treatment with an anti-ORAIP neutralizing mAb, indicating that ORAIP dominantly mediated the high glucose-induced apoptotic signaling. In addition, expression of ORAIP and apoptosis-induction by ORAIP in murine pancreatic β-cells strongly suggested that ORAIP plays a critical role in hyperglycemia-induced direct cardiac injury and pancreatic β-cell injury. In the present study, we used much higher concentration of (recombinant) ORAIP (10 mg/L) to induce apoptosis in pancreatic β-cells in the in vitro study than plasma ORAIP concentrations in DM rats (up to 300 μg/L) in vivo, as we did in the previous study using cardiac myocytes in vitro and myocardial ischemia/reperfusion in vivo [1]. ORAIP induces apoptosis in the cells as an extracellular ligand in an autocrine fashion. This occurs on the surface of the cells (such as cardiac myocytes and pancreatic β-cells) by binding of ORAIP molecules to their cell-surface receptors. Because cell-surface ORAIP molecules fall off into peripheral circulation or culture supernatants, the cell-surface concentrations of ORAIP (which we cannot measure) are thought to be much higher than those in peripheral circulation or culture supernatants. We used 10 mg/L of recombinant-ORAIP to induce apoptosis in cultured cardiac myocytes in the previous study [1], in which more than 70% cardiac myocytes underwent apoptosis in 72 h. This seemed to be an adequate concentration to analyze the apoptosis induction in vitro. Thus, 10 mg/L of recombinant-ORAIP is a standard concentration to induce apoptosis in cultured cells not subjected to the oxidative stresses. Apparently, there was no significant differences between cardiac myocytes and pancreatic β-cells in the susceptibility to recombinant-ORAIP (10 mg/L). Thus, ORAIP can be a novel biomarker and a critical therapeutic target for cardiac injury as well as pancreatic β-cell injury leading to irreversible progression of DM. Cardiovascular disorders are known to be frequently associated with DM patients, especially ischemic heart disease, supraventricular arrhythmias such as atrial fibrillation [18], and heart failure known as diabetic cardiomyopathy, which is characterized by disproportionate increase in ventricular mass and myocardial fibrosis [19]. These complications are mainly caused by diabetes-induced dyslipidemia as well as direct cardiac injury. Although many studies attempted to clarify molecular and metabolic mechanisms of diabetes-induced cardiac injury and reported multiple possible pathways involved [19], the precise mechanism has been only partially understood and is still elusive. For the mechanisms of diabetes-induced β-cell injury, several studies have reported the roles of intracellular mediators such as neuronatin, dynamin-related protein-1, hydrogen-sulfide, c-Jun N-terminal kinase, and ubiquitin/proteasome system [20,21,22,23,24]. Although these molecules may play a role in high glucose-induced pancreatic β-cell apoptosis, the strategy and effects of in vivo therapeutic intervention targeting these molecules seems to be questionable and unconvincing. Maedler, et al. [25] reported that Fas was upregulated in pancreatic β-cells in patients with type 2 DM and that high glucose induced Fas expression and apoptosis in human pancreatic β-cells, which was blocked by an anti-Fas antibody in vitro. This may offer anti-Fas antibody therapy against β-cell destruction in patients with poor blood glucose control, however, studies on the role of Fas in DM reported so far were limited to a T-cell-mediated autoimmune model, in which perforin rather than Fas plays a crucial role, and the role of Fas remains controversial [26,27,28]. Maier, et al. [29] reported that eIF5A depletion as well as the inhibition of hypusination protected against glucose intolerance in inflammatory mouse models of diabetes and that knockdown of eIF5A made mice resistant to β-cell loss and the development of hyperglycemia in the low-dose streptozotocin model of diabetes. The authors suggested inducible nitric oxide synthase (iNOS) to be the effector molecule downstream to eIF5A that mediates β-cell inflammation. At that time, they did not know that eIF5A can be hypusinated and tyrosine-sulfated, then secreted and plays a crucial role in apoptosis induction as a pro-apoptotic ligand in response to oxidative stresses [1]. Although the precise mechanism by which high glucose induces oxidative stress is unknown, the observation of Maier, et al. [29] strongly supported our findings in the present study. In the previous study of myocardial/ischemia reperfusion injury [1], we found that hypusination plays only a partial role in the ORAIP-mediated apoptosis-induction and that in vivo anti-ORAIP neutralizing mAb therapy markedly reduced myocardial infarction. Although in vitro data suggested that similar mechanisms were involved in high glucose-induced apoptosis in cardiac myocytes and pancreatic β-cells, further investigation is needed to confirm whether anti-ORAIP neutralizing mAb therapy can be useful against hyperglycemia-induced cardiac myocyte injury as well as pancreatic β-cell injury.
